# Simvastatin inhibits interferon-γ-induced MHC class II up-regulation in cultured astrocytes

**DOI:** 10.1186/1742-2094-3-16

**Published:** 2006-07-21

**Authors:** Esther Zeinstra, Nadine Wilczak, Daniel Chesik, Lisa Glazenburg, Frans GM Kroese, Jacques De Keyser

**Affiliations:** 1 Department of Neurology, University Medical Center Groningen, University of Groningen, Groningen, The Netherlands; 2 Cell Biology (Immunology Section), University Medical Center Groningen, University of Groningen, Groningen, The Netherlands

## Abstract

Based on their potent anti-inflammatory properties and a preliminary clinical trial, statins (HMG-CoA reductase inhibitors) are being studied as possible candidates for multiple sclerosis (MS) therapy. The pathogenesis of MS is unclear. One theory suggests that the development of autoimmune lesions in the central nervous system may be due to a failure of endogenous inhibitory control of MHC class II expression on astrocytes, allowing these cells to adapt an interferon (IFN)-γ-induced antigen presenting phenotype. By using immunocytochemistry in cultured astrocytes derived from newborn Wistar rats we found that simvastatin at nanomolar concentrations inhibited, in a dose-response fashion, up to 70% of IFN-γ-induced MHC class II expression. This effect was reversed by the HMG-CoA reductase product mevalonate. Suppression of the antigen presenting function of astrocytes might contribute to the beneficial effects of statins in MS.

## Findings

Currently available disease-modifying agents for the treatment of multiple sclerosis (MS) reduce the frequency and severity of relapses. They have to be given parenterally, are only partially effective, and are associated with adverse effects and high costs. An open-label clinical trial assessing simvastatin in patients with relapsing remitting MS revealed a significant reduction in gadolinium-enhancing lesions on magnetic resonance imaging of the brain, which is indicative of a disease-modifying effect [1]. Statins (HMG-CoA reductase inhibitors) are an attractive treatment option for MS because they are administered orally and have a relatively favorable safety profile. Clinical studies to test the effects of statins in MS are ongoing.

Statins reduce the migration of leukocytes into the central nervous system (CNS), induce a Th2 phenotype in T-cells, and decrease the expression of cytokines and inflammatory mediators [2]. A key step in the generation of autoimmune lesion formation in MS is the interaction of activated anti-myelin T cells with their specific antigen presented by major histocompatibility complex (MHC) class II molecules, expressed on the membrane of antigen presenting cells. Statins have been shown to reduce MHC class II expression in cultured microglia [3]. There is no consensus about whether microglia or astrocytes represent the principal CNS antigen presenting cells in MS [4]. A number of observations failed to detect MHC class II molecules on astrocytes in MS [5-7].

However, other investigators found that, in contrast to other conditions of CNS inflammation, scattered astrocytes at the edges of active MS lesions expressed MHC class II molecules [8-13], co-stimulatory B7 molecules [14], and adhesion molecules such as ICAM-1, indicating that these cells possess the necessary attributes to act as facultative antigen presenting cells [4]. We previously reported that astrocytes in the CNS of MS patients are deficient in β_2_-adrenergic receptors. We hypothesized that this defect allows IFN-γ released from activated T-cells to overcome the normal endogenous mechanisms that tightly suppress MHC class II expression on astrocytes [4,15,16]. In this study we assessed the effects of simvastatin on the interferon (IFN)-γ-induced upregulation of MHC class II molecules in cultured rat astrocytes.

Astrocytes obtained from neonatal Wistar rats were cultured in Dulbecco's modified Eagle's medium (DMEM) with 10% heat-inactivated fetal calf serum, 1% L-glutamine, 1% penicilline-streptamycine and 1% sodium pyruvate. A 95% pure astrocyte culture could be obtained. Cells were plated on coverslips coated with poly-L-lysine (PLL; Sigma, Saint Louis, MO, USA), until a monolayer was reached. All incubation experiments were performed 3 times in duplicate. To study the kinetics of MHC class II, IFN-γ concentrations of 6.5 × 10^-8 ^to 10^-12 ^were evaluated at 24, 48 and 72 hours. MHC class II expression in astrocytes was maximal following IFN-γ stimulation for 48 hours at a concentration of 6.5 × 10^-11 ^M (not shown).

Simvastatin at different concentrations from 10^-11 ^to 10^-8 ^M was simultaneously added with 6.5 × 10^-11 ^M IFN-γ for 48 hours. Cells were stained for MHC class II with mouse-anti-rat OX-17 (Serotec, Oxford, UK), 1:50 followed by secondary antibody sheep-anti-mouse biotin 1:200, 1 hour at room temperature, and incubation with alkaline phoshatase-streptavidin 1:300 for 1 hour. Blocking of non-specific background was done with 3% normal sheep serum. The coverslips were mounted in Aquamount. The percentages of positive cells were evaluated through microscopy and Quantimet image analysis (Leica, Rijswijk, The Netherlands).

We also performed immunofluorescence staining for GFAP and MHC class II with primary antibodies mouse-anti-rat OX-17 (1:25) and rabbit-anti-human GFAP (Sigma, Saint Louis, USA; 1:400) with 0.5% goat serum and 0.1% triton X-100, followed by secondary antibodies goat-anti-mouse FITC 1:200 and goat-anti-rabbit TRITC 1:400. Non-specific background was blocked with 2% normal goat serum. The cells were air-dried, coverslipped with anti-fading (DAKO, Carpinteria, CA, USA), kept in the dark, and analysed using confocal laser scanning microscopy. Semi-quantitative measurement of pixel density was performed with Scion image software (Scion Corporation, Frederick, MD, USA).

**Figure 1:**
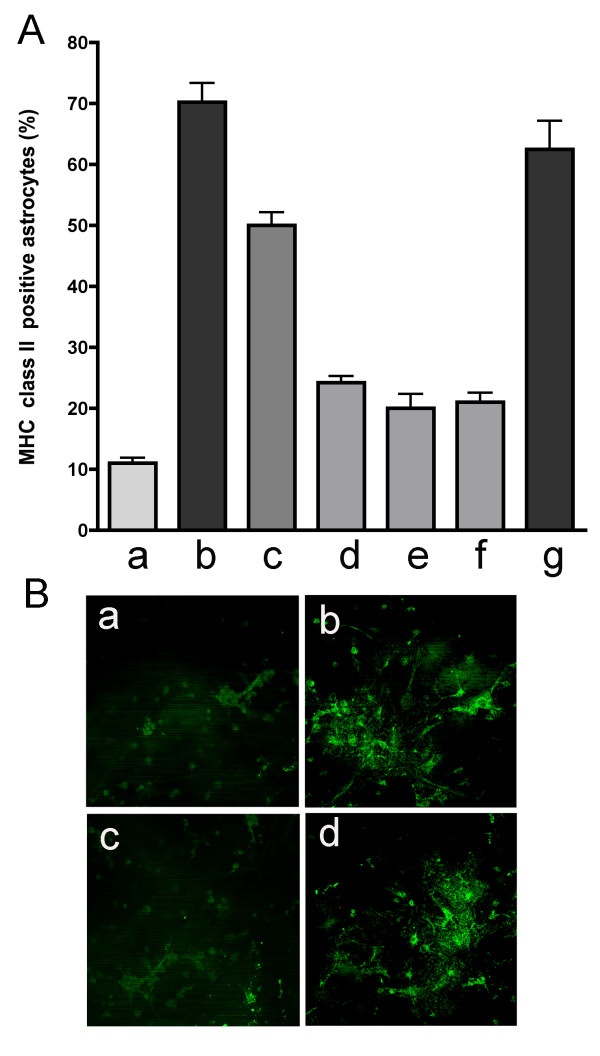
A. Percentage of MHC class II positive astrocytes (mean ± SEM). a, non-stimulated conditions; b, after IFN-γ stimulation, c, IFN-γ + 10^-11 ^M simvastatin; d, IFN-γ + 10^-10 ^M simvastatin; e, IFN-γ + 10^-9 ^M simvastatin; f, IFN-γ + 10^-8 ^M simvastatin; g, IFN-γ + 10^-8 ^M simvastatin + 100 μM mevalonate. B. Immunofluorescence staining for MHC class II. a, non-stimulated conditions; b, after IFN-γ stimulation, c, IFN-γ + 10^-8 ^M simvastatin; d, IFN-γ + 10^-8 ^M simvastatin + 100 μM mevalonate.

Results are illustrated in figure 1. In baseline culture conditions 11.0 ± 1.8% (SD) of the astrocytes expressed MHC class II molecules, with a mean pixel intensity of 55.0 ± 8.0 pixels/inch^2 ^as measured in the immunofluorescence staining. After IFN-γ stimulation, 70 ± 3.0% of the astrocytes expressed MHC II molecules, with a mean pixel intensity of 247.5 ± 9.5 pixels/inch^2^. Simvastatin 0.1 nM inhibited IFN-γ-induced MHC class II up-regulation by 70% (p = 0.004), and mean pixel intensity was reduced to 80.2 ± 12.9 pixels/inch^2 ^(p = 0.007). Higher concentrations of simvastatin (1 nM and 10 nM) did not produce greater degree of inhibition; about 20% of astrocytes remained MHC class II positive. These effects of simvastatin were reversed with the addition of 100 μM mevalonate (Sigma, St. Louis, MO, USA; 62.5 ± 9.3% positive cells and 236.3 ± 13.1 pixels/inch^2^), indicating that the inhibition of HMG-CoA reductase mediates the simvastatin-induced suppression of MHC class II on astrocytes.

IFN-γ-inducible MHC class II gene expression is transcriptionally regulated by class II transcriptional activator (CIITA). Astrocytic CIITA-deficient mice were resistant to experimental allergic encephalitis (EAE), an animal model of the inflammatory component of MS, although T cells proliferated and secreted Th1 cytokines [17]. These mice were also resistant to EAE by adoptive transfer of encephalitogenic class II-restricted CD4(+) Th1 cells, indicating that astrocytic CIITA-dependent MHC class II expression is required for CNS antigen presentation.

Simvastatin was only able to partially suppress IFN-γ-induced MHC class II immunostaining in astrocytes. Maximum effective inhibition was 70%, which is similar to that observed with interferon beta [18]. Whether this inhibition is also partial in vivo is uncertain, as MHC class II expression on astrocytes in situ is under control of endogenous inhibitory factors, such as norepinephrine, glutamate and vasointestinal peptide [4]. IFN-γ-induced MHC class II expression in astrocytes may partially be evoked by a mechanism that is not suppressible by simvastatin.

Statins have pleiotrophic effects on the immune system. The exact mechanism of action of statins in reducing disease activity of MS is uncertain. If the hypothesis that activation of astrocytes plays a key role in initiating autoimmune responses in MS is correct, then inhibition of astrocytic MHC class II expression may represent an important additional mechanism by which statins reduce disease activity in MS.

## Competing interests

The author(s) declare that they have no competing interests.

## Authors' contributions

EZ and JDK participated in the design of the study and prepared the manuscript. EZ, NW, DC and LG carried out the cell cultures, immunocytochemical analysis and quantification of the data. FGMK participated in the design of the study and helped to draft the manuscript. All authors read and approved the final manuscript.
